# 'Just have some IVF!': A longitudinal ethnographic study of couples' experiences of seeking fertility treatment

**DOI:** 10.1111/1467-9566.13429

**Published:** 2022-01-25

**Authors:** Ginny Mounce, Helen T Allan, Nicola Carey

**Affiliations:** ^1^ School of Nursing and Midwifery Oxford Brookes University/University of Oxford Oxford UK; ^2^ Department of Nursing, Midwifery and Child Health Middlesex University Middlesex UK; ^3^ Department of Nursing and Midwifery University of the Highlands and Islands Inverness UK

**Keywords:** couples, ethnography, fertility clinics, identity, in vitro fertilisation, infertility, IVF, longitudinal, normalisation, patient experience

## Abstract

We present findings from a longitudinal ethnographic study of infertile couples seeking treatment following initial GP referral to specialist fertility services. Repeated observations and interviews were undertaken with the same 14 heterosexual participants over an 18‐month period. Heterosexual, non‐donor couples comprise the majority of fertility clinic patients; however, research interest in this group has dwindled over time as IVF cycles have increased. In the United Kingdom, IVF is presented as a logical response to involuntary childlessness, and as an entirely predictable, and linear, course of action. The market is well‐developed and often patients' first experience of privatised health care in the NHS. Our couples were challenged by this, and while they felt expected to move on to IVF, some wished to explore other options. While IVF is ubiquitous, the discomfort and challenge around fertility treatments remain; experiences are prolonged and characterised by recursive narratives and expressions of disequilibrium, which are rarely acknowledged and reflected in ongoing clinic‐patient interactions. Our findings develop understanding of the process of ‘mazing’ (*Image ‐ The Journal of Nursing Scholarship*, 1989, *21*, 220), the pursuit of parenthood, by showing that the routine and normative status of IVF, at least in the current health care context, is at odds with the lived experiences of individuals.

## INTRODUCTION

Infertility has multiple medical, cultural and social meanings (Cousineau & Domar, 2007; Greil et al., 2011). We acknowledge that involuntary childlessness, fertility barriers or infertility episodes might be more inclusive of social or subjective considerations (Johnson et al., 2018) and, therefore, have used these terms throughout wherever possible. This contention of meaning is itself reflective of the complexities of the health care experiences we are examining in this paper; in the UK health landscape, the dominant biomedical framework conceptualises infertility as disease‐based, pathological or biological (Armstrong, 2002), even as infertility and treatments are social processes (Greil et al., 2011).

### Psychosocial effects

Societal changes, including contraception, delayed childbearing and feminism, have influenced cultural ideas about parenthood, and both men and women may choose to remain childless (Inhorn & Balen, 2002). However, given having a child remains socially desirable even in industrialised societies (Greil et al., 2011), the inability to conform to this social norm can be devastating and challenging for individuals. In strongly pronatalist settings (which advocate high birth rates), the social stigma of involuntary childlessness can lead to great suffering and economic hardship, especially for women (Inhorn & Patrizio, 2015). Being unable to intentionally conceive a child is acknowledged to be an extremely stressful life event (Dunkel‐Schetter & Lobel, 1991), and negative psychological repercussions may be severe for individuals and the social functioning of couples (Greil et al., 2011; Lalos, 1999). Both men and women are affected by involuntary childlessness and, although there may be gender differences in relation to its impact and treatment (Culley et al., 2013a), it can be perceived as a crisis by both (Greil, 1997; Menning, 1975). Typically, it produces biographical disruption (Bury, 1982), with reactions similar to that of grief and mourning (Waltkins & Baldo, 2004). The issues associated with infertility are complex, and the experiences of individuals' seeking or embarking on treatments are personal and varied. Attempts to become parents precipitate ‘a life‐changing journey’ that can last months or years (Shapiro, 2009, p.143).

Often many individuals and couples attempt to resolve the disruption of infertility within the prevailing biomedical model, so shifting understanding of reproductive failure as a mainly social problem (childlessness) to a medical one (infertility) (Becker & Nachtigall, 1992). From the 1970s, treatments have involved assisted reproductive technologies (ARTs) such as in vitro fertilisation (IVF). In the United Kingdom, significant numbers of babies, around two per cent, are born each year following IVF (HFEA, 2020) with typically three or more cycles needed to obtain a pregnancy with live birth (Smith et al., 2015). These treatments are known to be highly stressful for patients and may add to the negative experiences of continued involuntary childlessness (van Balen & Trimbos‐Kemper, 1993; Domar et al., 2018; Peddie et al., 2005). Our research question was ‘what is the experience of starting investigations and treatment like for couples who have failed to conceive?’.

### Assisted reproductive technologies

ARTs are important financially (Blakely et al., 2019). Globally, accelerated growth in IVF volume is reported (LaingBuisson, 2018) and its business model has penetrated both commercial markets and medical sectors. Infertility is now viewed as something which should and can be treated and it is IVF, once subject to intense media scrutiny that increasingly appears as a routine treatment (Allan, 2009; Franklin, 2013). However, it is worth noting that seeking (IVF) treatment, at least in the United States and United Kingdom, is a predominately white, middle‐class undertaking (Becker, 2000; Jain & Hornstein, 2005). The treatment seekers in clinics are, therefore, often not representative of all and rarely include minority ethnic populations (Culley et al., 2007).

Kamphuis et al. (2014) describes an over reliance on IVF; intra‐uterine insemination could be a safer, cheaper and more acceptable option for some (Bahadur & Homburg, 2019), and less invasive techniques could improve access to ARTs. IVF remains expensive, and funding of IVF treatment is rationed in the United Kingdom. Because of fairly low rates of ‘success’ (HFEA, 2020), multiple IVF cycles are often required, and the majority of the financial burden falls on patients. Medical consumerism is now understood as part of the NHS landscape (Mold, 2010), but patients are not always aware that services such as IVF, social care, some aspects of primary care may require payment. Additionally, long‐term safety using frozen embryos, which accounts for over half of all UK IVF cycles (HFEA, 2020), is uncertain (Berntsen et al., 2019, Maheshwari et al., 2016). Consequently, IVF is not a panacea, neither is it routine (Allan, 2009).

The experiences of involuntary childlessness, ARTs including IVF and new forms of family building (Gürtin & Faircloth, 2018), continue to be of interest to researchers. Heterosexual couples' experiences of non‐donor IVF have received less attention, despite evidence that ARTs in this group continue to shape experiences of pregnancy and early parenthood (Allan et al., 2019, 2021). We focus attention on heterosexual couples seeking parenthood, who make up the majority of fertility clinic patients, and argue that despite IVF's seeming routinisation, their experiences remain worthy of our regard.

## AIM

Our longitudinal study was designed to problematise the acceptance of IVF as a routine medical treatment. We aimed to investigate the experiences of couples as encountering the biomedical framing of their situation in fertility clinics. Couples' experiences around this time are currently not well understood, although it is known that many seeking medical help for fertility drop out before starting treatments (Passet‐Wittig & Greil, 2021).

## METHODS

A longitudinal ethnography was designed to answer the research aim. All couples who were booked to attend a single‐site NHS fertility clinic in a 3‐month period, following a primary care referral, were sent a study invitation letter, a participant information sheet and prepaid reply slip to indicate whether they were prepared to discuss the study. Those who did were contacted at this appointment by the researcher, and if they agreed to take part, written consent was obtained. Verbal confirmation was reiterated at each subsequent study contact. From the 50 letters sent, fifteen couples replied and all were contacted. Fourteen were recruited and participated in the study. All the couples were heterosexual, although this was not a study inclusion criteria (Table 1).

**TABLE 1:** Characteristics of participants

Pseudonyms	Age^a^	Ethnicity & Couple status	Occupation	Fertility background
Adam/Eve	32/29	White, British married	Business/marketing	Never pregnant, male factor
David/Victoria	51/41	White, non‐British married	Education professionals	Never pregnant
Mark/Virginia	35/35	White, British co‐habiting	Self‐employed/business	One child
William/Katherine	37/40	White, British co‐habiting	Self‐employed	Never pregnant
John/Thea	32/29	White, British married	Legal/other professionals	One child, following Clomid
Steve/Rose	31/30	White, British married	Service/business	Never pregnant
Dev/Sara	41/40	White, British married	Managerial/professional	Never pregnant
Doug/Erica	32/31	White, non‐British married	Scientist/service	Never pregnant
Karl/Janet	37/38	White, British married	Military/service	Never pregnant
Andy/Cath	36/35	White, British married	Self‐employed/service	One child
Ali/Becky	34/34	White, British married	Military/business	No children, miscarriage
Arley/Caroline	36/36	White, British married	Business/educational	One adopted child, miscarriage
Tony/Elaine	35/34	White, British married	Education professionals	Never pregnant
Nick/Debbie	36/36	White, British married	Medical professionals	Never pregnant

^a^ Age at first study contact.

Data collection involved repeated rounds of participant observation and interviews with the same participants over a period of 18 months (March 2013–September 2014) at their first NHS clinic appointment and thereafter for any subsequent appointments or treatments. Participants referred on for fertility care were seen at one privately run fertility clinic. All patients seen in this clinic were either funded for one (occasionally more) cycle of IVF, depending on criteria set by their local commissioning group, or were self‐funding (Table 2).

**TABLE 2:** Study participant contact dates

ID	Pseudonyms	Contact date and event									
C1	Adam and Eve	3/13/2013 observation	3/19/2013 phone call								
C2	David and Victoria	5/13/2013 observation	5/20/2013 interview	6/12/2013 phone call	8/28/2013 phone call	10/7/2013 observation	10/11/2013 phone call(s)	12/1/2013 interview	2/20/2014 observation	2/28/2014 phone call(s)	9/1/2014 interview
C3	Mark and Virginia	5/20/2013 observation	7/1/2013 interview	7/10/2013 email	9/2/2013 phone call						
C4	William and Katherine	6/3/2013 observation	6/20/2013 interview	11/29/2013 phone call	1/10/2014 phone call(s)	5/1/2014 interview					
C5	John and Thea	6/3/2013 observation	6/13/2013 interview	9/16/2013 observation	10/25/2013 phone call	11/5/2013 phone call	11/19/2013 interview	1/29/2014 observation	3/19/2014 phone call		
C6	Steve and Rose	7/31/2013 observation	8/5/2013 interview								
C7	Dev and Sara	9/23/2013 observation	10/2/2013 interview	10/11/2013 phone call(s)	10/29/2013 observation	12/5/2013 interview	2/4/2014 observation	2/18/2014 interview	3/28/2014 observation	4/2/2014 contcact(s)^a^	4/25/2014 interview
C8	Doug and Erica	11/11/2013 observation	11/12/2013 email/calls	12/1/2013 interview	3/12/2014 Contact^a^	8/26/2014 observation					
C9	Karl and Janet	1/6/2014 observation	1/7/2014 email	1/13/2014 email	2/7/2014 interview						
C10	Andy and Cath	1/7/2014 observation	1/7/2014 text(s)	1/29/2014 observation	2/17/2014 interview	5/29/2015 text(s)					
c11	Ali and Becky	1/9/2014 observation	1/9/2014 email	1/22/2014 interview	3/10/2014 observation	3/20/2014 interview	3/24/2014 text	5/6/2014 contact(s)^a^	7/10/2014 interview		
C12	Arley and Caroline	1/28/2014 observation	2/3/2014 phone call	2/20/2014 interview	3/26/2014 observation	5/29/2014 text	6/16/2014 interview				
C13	Tony and Elaine	1/30/2014 observation	2/19/2014 interview								
C15	Nick and Debbie	2/17/2014 observation	2/25/2014 interview	4/2/2014 observation	9/25/2014 email						

^a^ ‘Contact’ is brief contact such as conversation at clinic.

Data were collected through participant observation in the clinic setting by the first author (GM) recording field notes, reflections and observations, using a notebook and dictating into a digital recorder. Observations were made of all couples' first appointments at a hospital fertility clinic, and other follow‐up consultations, for example, ad hoc observations of clinical events such as nursing appointments and other procedures, including those couples that had IVF treatment. Other sources of documentary data included field notes, conversations with clinic staff and a research diary. Couples were interviewed together in their own home or clinic space by (GM) for up to 90 min following each consultation. The interviews were recorded and transcribed verbatim by GM with pseudonyms replacing individual names and other identifiable data. In the findings, ‘we or us’ refers to the authors although only the first author collected data. As an insider in the clinical setting, working as a research nurse, she was frequently present and familiar with the staff, resources and organisation of the clinics.

It is important to consider GM as research nurse here. In the IVF clinic for example, the HFEA view is that a research nurse should be available to offer independent advice to patients considering taking part in research studies, while also recruiting these patients to these same studies. When the research nurse is also conducting the data collection for her PhD (as in this case), especially when using participant observation that entails her prolonged presence in the clinic, the role takes on the classic insider/outsider duality of ethnography (Hammersley & Atkinson, 2007). The research nurse simultaneously becomes part of, by virtue of being a health care professional, and separate to, as a PhD candidate, the culture of other nursing and medical staff in particular health care settings (Hunt & Symonds, 1995). This duality and carefully negotiated positioning is a particular challenge. It involves negotiation between the dual roles to maintain the cultural separation of both through professional identity such as appearance, attitudes and behaviours. PhD data were, for example, collected on separate days to her research nurse days, and she wore no uniform which marked out her different role to both colleagues and patients. Rather than approaching a number of patients on a shift, she would accompany one couple during an observation. Patients appeared to recognise the field relationship for what it was, that is, of a researcher rather than a clinic staff member, and although the content of interactions was intimate, the relationship itself did not become so. However, although participants themselves appeared to behave unselfconsciously, the clinic staff were more clearly aware of the researcher's presence in a different role to her accustomed role. They would include her in conversations or ask her opinions. The researcher maintained distance in these situations through active awareness and body language, retained existing role boundaries and never discussed the research participants with staff. Throughout participant observation, the researcher kept reflexive notes on her presence in the social world being studied, and used this to promote critical reflexive practice at all stages of the data collection, analysis and reporting. Adopting a reflexive attitude, supplemented by regular research supervision, diaries and journals, was a large part of the fieldwork and in the development and creating meanings (Hammersley & Atkinson, 2007).

All couples had at least one consultation and interview. In Table 3, their study outcomes (when they finished their participation in the study) are shown. This varied from completing a tertiary treatment cycle, such as IVF, becoming pregnant or deciding not to continue with investigations or treatment at any point.

**TABLE 3:** Outcomes of study participants

Couple ID	Pseudonyms	Study outcome
C1	Adam/Eve	Withdrew after consultation
C2	David/Victoria	Stopped before planned IVF
C3	Mark/Virginia	No treatment planned (after first consultation)
C4	William/Katherine	Pregnant naturally, pre‐IVF
C5	John/Thea	No further treatment planned
C6	Steve/Rose	Future IVF planned
C7	Dev/Sara	One cycle IVF completed
C8	Doug/Erica	No treatment planned (after first consultation)
C9	Karl/Janet	Future IVF planned
C10	Andy/Cath	No further treatment planned
C11	Ali/Becky	One cycle IVF completed, pregnant
C12	Arley/Caroline	Clomid cycle, pregnant
C13	Tony/Elaine	No treatment planned (after first consultation)
C15	Nick/Debbie	Clomid cycle

The longitudinal design enabled data collection over an extended period (Murphy‐Black, 2000) to allow understanding of the couples' experiences over time. This approach is useful in investigating health care processes (Grossoehme & Lipstein, 2016) and adaptations to events. The long time period resulted in a huge quantity of data, captured as language and text. Data analysis used an adapted model of thematic analysis (Braun & Clarke, 2006) based on accounts of interpretative phenomenology (Ajjawi & Higgs, 2007; Smith, 1998), which sought to identify couples' and cultural meanings within the data as opposed to a solely idiographic focus. Strategies such as the use of reflexivity and the active role of researcher were used to analyse the data to allow meaningful interpretations. This meant themes did not ‘emerge’ from the data but were judged as being important to the research question; in this sense, the analysis was interpretative. The data were not analysed with a view to identifying items prevalent across the data sets. The analytic process was iterative, with examination of underlying ideas, assumptions and ideologies from both the literature, the researcher herself and ideas which were discussed during supervision (Braun & Clarke, 2006). Analysis ran concurrently with data collection during the longitudinal period, in a classic ‘hermeneutic circle’, moving to and fro between the emerging interpretations made by the researcher and ongoing examinations of the textual data (Ajjawi & Higgs, 2007). Themes were refined to be descriptive of experiences (Smith, 1998). The final analysis was to consider the overall themes and whether these had changed over time. During data analysis, metaphors were intuitively selected as means to illuminate accounts of participant's experiences, but in the end they were felt to be insufficiently sensitive to represent individual accounts, and so were not used as final descriptors. However, as discussed later, the ‘journey’ metaphor was one retained as ubiquitous in clinicians' and participants' talk in the clinics and in interviews. This led to a re‐exploring of this familiar metaphor to allow meaningful, fresh insights.

Data validity and credibility were demonstrated by the use of triangulation through multiple sources of data collection (Greenhalgh & Taylor, 1997), ongoing member checking, supervisor team reading and rereading transcripts and analytical write‐ups, and prolonged involvement in the field. The quality of verisimilitude of the findings was confirmed several times by audiences at seminars and talks.

The study was approved by a NRES Research Ethics Committee (REC 12/SC/0571). Pseudonyms are used for all individuals throughout.

## FINDINGS

Main themes were constructed following data analysis: becoming a fertility patient; choice and commercialisation; and mazing revisited. These themes position the experiences of heterosexual couples seeking fertility treatments in the United Kingdom within the landscape of the mature IVF marketplace described in the introduction.

### Becoming a fertility patient

The theme of ‘becoming a fertility patient’ is primarily about couples' reconciliation to assuming a medically assigned patient role which caused considerable tensions for the majority of couples. Our data confirmed several aspects of fertility treatment seeking that are already known yet nonetheless important and enduring features of the medicalisation of infertility among patients who seek medical treatment. These latent themes, or underlying ideas, included: descriptions of imagined futures with children; the dawning realisation of challenges ahead; some couples spending years *trying* to conceive; the negative feelings and uncertainty they experienced while trying to conceive; the upset to their imagined future life courses and negative effects on previously taken‐for‐granted gendered roles that involuntary childlessness caused.

In our study, without exception, the doctors began their consultation by addressing the female partner. However, male partners were emotionally engaged and in our data some men wished, even more than their partner, to attend to the causes of their failure to conceive and were observed actively seeking more attention towards themselves during consultations. In arranging and attending the first consultation, couples engaged with medicalisation discourses, practices and spaces, including those outside the hospital such as newspaper articles and online sites. Some couples had positively reinterpreted their own situation in the light of this engagement; they felt themselves ‘ready’ for or ‘appreciative’ of parenthood compared with those who ‘just fall pregnant’ (Caroline). This positive framing of ‘trying’ for parenthood (using assistance) may be the result of the biomedicalisation of ARTs and their normalisation in popular discourse but is in contrast to other studies which show that involuntarily childless couples feel angry towards couples who conceive (seemingly) easily. However, even those who had been hopeful, or able to frame their fertility more positively, found themselves fragile in the face of waiting to progress along the medical fertility pathway.

Couples were enrolled into our study at their first appointment after GP referral, and while, as Nick suggested, ‘we are not ill’, this appointment included the discomfort of receiving a diagnosis or as Erica described it:

[you] kind of ‘bury it’ deep inside so that it doesn't affect you, and then it's just that someone brings you to the surface and shows it to your face and tells you. (Erica)

This meant facing a medical assessment and intrusive enquiries into their lives when they felt otherwise well. For all our participants, a diagnosis was personal and provoked discussions of individual fault and blame. As already known, the language used in medical encounters is not received neutrally and serves to reinforce the negative feelings around infertility (Silva & Machado, 2008). At the same time, diagnosis was important for legitimising their condition and suggesting it might be remedied, although a significant proportion had received the disconcerting diagnosis of ‘unknown’. The diagnosis in most cases, even if inconclusive, led to treatment with ART. The diagnosis ‘infertile—for IVF’ was a surprise to most of the participants who had expected more investigations and did not expect to be told at the first consultation in the fertility clinic that their most likely course of therapy was IVF.

Although there was some reluctance in acknowledging their status as *fertility* patients and in the rapid nature of diagnosis and treatment planning which was sometimes felt abrupt (‘there you go, that's what we think, go and get on with it!’ (Cath)), most couples had also invested emotional capital in their first appointment. They expressed their desire for action being ‘almost excited’ and ‘keen to get on with it’ (Adam and Eve), and they were ‘happy’ (Rose) or ‘relieved’ (Thea) to have their consultation at the hospital clinic.

After this appointment, the impetus for treatment mainly resided with the couples and, in most of the consultations, to consider IVF. The couples had the decision to go ahead and self‐refer to an IVF clinic. While driven by the desire for parenthood and warned of the limited biological time available, this still did not necessarily translate into particular urgency among the couples to take the next step, that is, start the IVF referral process. For example, Victoria recalled ‘dragging my feet’ in the hope that it might ‘happen naturally’ and Doug told a story of friends who ‘suddenly at some point—at 50—they had a child!’ These types of hopeful stories were repeated by several couples throughout our prolonged contact with them, even, poignantly, when they had decided to stop treatments.

### Choice and commercialisation

As described, typically the first consultation included an infertility diagnosis and treatment recommendation, such as investigative surgery or, if appropriate, ovulation induction, and generally some form of IVF. Appointments were no longer than 30 min and were process‐driven, with little time for discussion of feelings. Any emotions both men and women expressed at the consultation were infrequently addressed, and the impression of ‘you're another number in my day’ (John) was a common finding. It was assumed that couples would inevitably ‘move on’ towards IVF.

I wasn't expecting the kind of black and white … basically try IVF or try your luck, kind of thing (Debbie)

It was at the first consultation that the apparent ubiquity of IVF became clear to couples. Clinic materials and information all described IVF, and consultants invariably introduced and considered IVF a possibility. For example, Victoria said:

I just … feel like there's also steps that have potentially been not taken into account or if they're not viable steps he could have gone through why they were not viable to get us to the IVF option, rather that starting out with that

Virginia felt it seemed to be:

Just have some IVF! Yeah! Just have it. Yeah it did all seem a bit … it's fine for him to say but he's not the one that's going, that's going through it.

Becky said that she had been ‘hoping that [IVF] would be what would be suggested’ but her partner was more cautious:

A lot of people think it is just going to work straight away, when I've told a couple of friends and I think our family … and I think because we have had friends, none of them worked first time and so we know that it's not just ‘click your fingers’ and the first time it happens. So, I think that's a funny thing to get around isn't it, people just go, ‘Oh you're having trouble, just have IVF!’ and not realise what a big deal it is. And how long it can take (Ali)

Decision‐making was important as Nick explains:

It really does give us two choices, which is to have quite significant interference with nature or something else … but … adopting or not having … things which we semi‐talked about, a bit, but haven't really … not really in a position to decide that in our minds.

Some were unable to opt for IVF treatment, even if this is something that, after considering all the alternatives, they would otherwise choose to do, because they did not have the financial means:

There was no financial way for us to actually go for IVF in the near future anyway (Arley)

We don't have that kind of money sat in the bank (Cath)

Although staff were not interviewed, one or two of them wanted to talk after being observed. From what was said after consultations with the researcher, clinicians were aware of the significance of the consultation for patients. One doctor remarked that a consultation had been ‘quite easy as she [the doctor] was able to offer a solution [IVF]’. She went onto say ‘that when there was nothing else [no treatment to offer] or money was an issue then this was more tricky’. She said she had lots of tears last week with all her couples, and this made her wonder ‘what am I doing?’ (Dr L).

Couples unable to pay for at least one cycle had little option but to ‘wait’ for some unspecified further help (although, two participants later fell pregnant naturally). Those who continued with the option of IVF found the distinction between clinical advice and marketing of treatments to be unclear and found the overtly business aspect of IVF disconcerting:

I was literally sat there and watched the finance man come out, take somebody in for payment and I saw her come out with her receipt and wrap it round her credit card and put it in … and I thought this was like a business, it didn't feel like a ‘baby place’ (Cath)

Couples were suspicious of the juxtaposition of public (NHS) and private health care:

A: … and I thought I'm pretty sure **we** won't be seeing Doctor X today!

B: [laughing] we were like, ‘they must have paid!’ [all laughing]

A: yes, on the gold card! (Becky and Ali).

The uncertainty and emotions that men and women describe above continued throughout the subsequent interviews and observations which lasted for many months for each couple. Becky explained ‘There's the only so much you can do. There's only so much you can control, so there's no control’ while for Sara the ‘uncertainty’ and ‘anticipation’ made her ‘more fearful’, ‘worse’ and ‘terrified’. This longitudinal, iterative data collection and analysis was particularly important in observing the lengthy nature of the infertility journey and continued disequilibrium felt by couples during their time in contact with the clinics. Their uncertainty and shock at their experiences, particularly in relation to the centrality of IVF in consultations, was at odds with the ways in which IVF was reinforced at every medical encounter.

Our final theme considers how the theory of mazing, which includes the work couples do in adapting to their situation, is still relevant where IVF has become a ubiquitous, routinised and normative process.

### Mazing revisited

The examination and re‐examination of issues that preoccupied couples as they try to make sense of their infertility was described over 30 years ago as ‘mazing’ (Sandelowski et al., 1989), a recursive and intenseful effort in the seeking of parenthood. Our findings show that couples engage in mazing as circuitous discussions back and forth between partners and between the couples and the staff at the clinics. These discussions reveal their continued uncertainty about the apparent lack of choice and reliance on IVF. Examples include Virginia revisiting the same issue about her previous gynaecological surgery several times in our interview, David and Victoria ruminating on their discussions about IVF and Thea's long description of her reasons for remaining unconvinced by what a consultant had told her. The tension between the desire for the treatment pathway to be linear, and the ubiquity of as IVF, was reflected in repeated expressions like: ‘forward’ ‘move on’ and ‘get on’ along with couples saying, as Mark did, ‘how we move on from here really… left him … confused’. Erica's comment illustrates some of the tension and disquiet that the proposition of IVF invokes:

It's not really how I want to have a baby, I wanted it to be natural so … yeah [pause], I am not really ‘into it’.

The routinisation was reflected in clinic interactions and mainly led by treatment processes, timetabling and plans. Couples had to ‘fit‐in’ treatments around their own work schedules, but there was little flexibility available for them to do so. Sometimes comments were made when patients made requests for different appointments or phone calls at a particular time (‘apparently she's got a really important job!’) or when men called on behalf of their partners. This echoes previous findings of nursing care in fertility clinics (Allan, 2001) where routine replaces compassion. However, active treatment phases of ‘scans every day’ or ‘calls every day’ offered couples some relief, although the questioning continued, often oscillating between ‘fear’ and hope (‘you never know’) for their future.

For one of our couples, mazing as a continued uncertainty and questioning ‘put a strain on our relationship which was not helpful’ and precipitated their decision not to start IVF treatment. Nevertheless, and previously discussed as a common feature of fertility patients, despite deciding not to pursue IVF, they continued to be hopeful for their own future:

It seems loads of people go through it and have IVF and for some it works and for some it doesn't … and one friend, A‐‐ had IVF, then took a long time, they thought they'd try naturally, had a drunken evening, were about to start IVF again and she found out she was pregnant. And then had a third naturally. (Victoria)

Another aspect of the routinisation of IVF treatment is seen when a ‘cycle’ is completed. Adjusting to negative outcomes or stopping treatment was very hard for couples to accept as final; two of the couples in our study, who had decided to end treatment, much later continued with further cycles of IVF using donor gametes.

## DISCUSSION

Involuntary childlessness causes great suffering (Lalos, 1999) and long‐term emotional turmoil yet its impact is downplayed by society as a whole (Brian, 2011) and popular cultural commentary can be judgemental (Widdecombe, 2013). Previous research has investigated patients’ experiences in the earlier days of infertility investigations (Sandelowski et al., 1989) when ARTs and IVF were *emerging* technologies (Becker, 2000; Imeson & McMurray, 1996). This earlier work largely focussed on women, even in those studies which included men (Culley et al., 2013a). Meerabeau's ethnography of couples attending British National Health Service (NHS) fertility clinics (Meerabeau, 1995) and Allan's ethnographic study on the management of emotions within the fertility clinic setting (Allan, 2001) were among a small number which used observation to understand how patients experience the biomedical process of IVF in hospital systems. Significant research on the psychosocial aspects of infertility (for example Boivin et al., 1998) examined social psychological effects, but the ethnographic, longitudinal nature of our study, suited to the ‘journey’ concept, with a lens on the routinisation of IVF, is unique.

Franklin (2013, p749) describes IVF as paradoxical, being both a ‘normal and regular fact of life’, as represented to society at large and by the millions of babies born, and simultaneously ‘not’ for those individuals contemplating it. In clinics there is an expectation to use this technology and for patients to demonstrate a willingness to try (Toscano & Montgomery, 2009), even if the hope is frequently belied by outcomes (Jardine, 2013). Despite its ubiquity in clinic settings, however, beliefs about IVF in the wider population of reproductive age adults vary (Weston & Qu, 2005). Just under half of European survey, respondents say they would consider IVF if needed (Fauser et al., 2019). Our participants had usually spent many months, if not years, deciding whether to go ahead with treatment, and they often appeared unable to make decisions quickly. The clinics did not help in this regard; there was little opportunity for neutral discussions to help decision‐making, and the clinics themselves provided the diagnosis and information sources, such as from the HFEA, which patients use to aid their choices. IVF clinics can seem fairly blasé with statements such as one by a senior fertility nurse of a large UK clinic who said ‘It isn't starting fertility treatment that's hard… it's stopping it, and knowing when to stop it’ (Moorhead, 2015). All this implies that while, as we have argued, IVF is now the dominant model for medical fertility treatment, it remains a troublesome concept, at least for individuals, and our findings underline this.

Couples, even where they had access to straightforward or funded treatments, expressed regret at being fertility patients at all in a rejection of the infertile label, retaining a wish that they would conceive naturally. They questioned health care professionals and made continued enquiries of the internet and their friends during the time they were in the study; that is, during the entire time they were in contact with the fertility clinic. This suggests that the information given out by the clinic was inadequate either because it did not meet their needs; or because the information was not critical enough and did not capture their ambivalent feelings towards IVF. Medical treatments on offer were presented in clinic consultations and in patient information as legitimate knowledge based on the scientific and technical, there was very little discussion of lay knowledge. This reinforced the stigma of infertility (Allan, 2017) by emphasising it as a problem to be fixed by experts. Our findings reinforce ideas that help seeking is complicated by these opposing positions and couples’ reconciliation to a role as ‘fertility patient’ involves what Thompson describes as ‘ontological choreography’ (Thompson, 2005). It is known that many couples do not pursue treatments, even if they have made initial enquiries (Passet‐Wittig & Greil, 2021) and underlines our findings that ‘becoming a fertility patient’ itself is not straightforward, but not just because of a conflict of identities. The biomedical model of IVF assumes newly diagnosed patients are willing to undergo IVF, but we show this sits uncomfortably with couples and many were unable to dissociate entirely from the largely unscientific hope and emotion they had already invested in their own care (Meerabeau, 1998).

Sandelowski et al. (1989) theorised that couples investigated and sought solutions to achieve biological parenthood by negotiating their way through a maze of alternative actions. When her paper was written, IVF was still a new and developing technology; now those options are no longer presented as alternatives for most couples (Bhattacharya et al., 2008; National Institute for Health and Care Excellence (NICE), 2013); instead IVF is offered routinely. Nevertheless, the imagery and descriptions of the ‘labyrinthine’ processes involved in seeking solutions are evocative of the effortful quality and time that these negotiations require from patients even when the choices are narrower than 40 years ago. Our findings show that these processes are still usefully described as a journey, albeit one with many twists and turns. The struggles still experienced by patients echo the previously identified processes couples undertake in managing their infertility identity (Olshansky, 1987) although this is further complicated by great variations in self‐identification by individuals who seek fertility treatments (Leyser‐Whalen et al., 2018).

Through the repeated contact with participants, we saw that this turmoil was largely unacknowledged in any contacts with clinics or staff, despite being a key feature of their experiences. The unique longitudinal design of our study enabled us to ask repeat questions of our couples as they had further medical encounters. These repeated contacts served to underline that the demanding and recursive qualities that characterise their experiences (Cipolletta & Faccio, 2013) were not relieved during treatments nor consultations. Nor was their medically ascribed label, ‘infertile—for IVF,’ unproblematic.

Worldwide, IVF has enabled biological parenthood for many people. However, couples were surprised by IVF's ubiquity and they were disappointed by a lack of other alternatives or treatment choices; the possibility of IVF caused them further uncertainty. Our findings add to the existing literature of the demands IVF places on patients (Hammarberg et al., 2001), by demonstrating that while clinics simply expect couples to ‘move on’ to IVF, reinforced by its linear presentation (Department of Health, 2018), couples themselves want to explore other options but find there are none. The clinic–patient relationship is now further complicated in the United Kingdom due to the semi‐privatisation of NHS fertility clinics and mature IVF marketplace, where patients are encouraged to behave like consumers (Franklin, 2013). While this has been the case across the NHS more broadly, the patient experience of commercialisation, commodification and marketisation of the NHS has largely been hidden (Sturgeon, 2014). As Mold (2010) argues, patient demand for choice historically was instrumental to the introduction of the pseudo‐market into the NHS. Thus, patients may feel their ability to choose will lead to greater access within a funded NHS and are surprised and disappointed when this choice is unsubstantiated (Allan, 2016). Our participants were also disappointed by the care they received which, in spite of the high fees, attractive clinic spaces and emphasis on reproductive science, often left them in a distressed state with an uncertain future. The transactional nature of the relationship was in stark contrast to the promotional materials, which promised much more. In America and Australia, commercialisation of ‘boutique’ fertility clinics started earlier than in the United Kingdom (Allan, 2009; Thompson, 2005), although this model has been criticised (Blakely et al., 2019). Allan (2016) suggests patients find it difficult to untangle the advice that they are being presented with because they are simultaneously being introduced to and treated within a marketised system.

The negotiations inherent in these models are one explanation for the inequality of access to treatments (Bell, 2010, 2014), financial cost being another, reflected in the homogeneity of clinic patients (HFEA, 2019). In the United Kingdom, IVF funding is rationed and the majority of patients have to self‐fund treatments (HFEA, 2020), frequently multiple times (Smith et al., 2015). It is this cost which prevents some patients from utilising IVF (Connolly et al., 2010), but it is not until couples reach the fertility clinic that they realise this. However, the disquiet expressed by our couples at the financial aspects is perhaps more to do with the realisation of what this makes plain, that is, the transactional (and ubiquitous) process of producing a baby that IVF involves.

Lack of emotional support and continuity of care have previously been identified as weaknesses in the delivery of fertility care (van Empel et al., 2010). It is apparent that ‘couples are struggling’ but little is done to help them in any meaningful way. Fertility nursing staff have extended roles in clinics (Allan & Barber, 2004) with the potential to offer this continuity and support to patients (Payne & Goedeke, 2007) although the necessarily ‘emotional engagement’ is sometimes missing, with the nursing role seen to be managing the clinic rather than the patients (Allan, 2001). There are European sector guidelines (Gameiro et al., 2015) which recognise the value of routine psychosocial care provision, but our findings demonstrate that for some individuals at least, it is this routinisation of care in clinics which is the issue. The clinic bypasses the obvious vulnerabilities of couples, takes no interest in it and makes no room for it, but expects them to fit in and conform. All of these things only add to the couples' feelings of grief and loss, and they continue to struggle.

Finally, the conceptualisation of the fertility ‘journey’ implies a treatment process that propels couples towards a certain outcome (successful or not). The use of the metaphor ‘journey’ is of course a cliché, but is commonplace amongst the users, staff and organisations of fertility services, as an easily understood description of a complex process. Numerous examples exist, from company websites, laboratory materials, brochures and patient accounts (see for example HFEA, 2021). Participants used the journey metaphor (also alluding to travel, paths, destinations) to illustrate or to explain their experiences. That more literal language might be inadequate for this purpose (Palmer‐Wackerly & Krieger, 2015) is telling and emphasises how un‐routine fertility treatment is.

The locus of pursuit that Sandelowski et al. (1989) described does not end with treatment discontinuation, nor do all patients completely accept their status as infertile (Peddie et al., 2005) at least for an unknown time. Some feel that the effects of unwanted childlessness never completely goes away even if they become parents (Allan et al., 2019; Brian, 2011), echoing earlier work (Olshansky, 2003; Sandelowski, 1995) suggesting patients retain an infertile identity which may continue even into their parenthood. However, not all clinic patients self‐identify as infertile (Leyser‐Whalen et al., 2018) and may, therefore, not recognise this as a consequence of treatment for themselves. Our findings show that couples retain their indeterminate state at all stages of the ‘fertility journey’, even following failed cycles or decisions to end treatments, as they were seemingly unable to stop hoping for a child or for more treatment. The routinisation of IVF as a catch‐all treatment belies the significance of infertility on couples' lives and masks the great demands and effort required by them.

## CONCLUSION

The concept of ‘the fertility journey’ portrays IVF as a smooth course of events, based on scientific evidence, leading to conception and birth. Re‐exploring the concept of this journey, we show, through sustained contact with couples, that this experience is a long, effortful undertaking, with continued questioning and re‐examination of self and purpose, which is often left unresolved by treatment process. The commercialisation of the sector has introduced market pressures which are not helpful for patients; it adds to the inequalities of provision and take‐up. This was a small‐scale study, with data first collected 5 years ago. However, the longitudinal design and methodology resulted in findings of depth and provided insight into the experiences of the participants which we argue remain true to clinical practice and patient experience today. The findings are not generalisable, although commonalities of experiences were revealed. The sample was typical of the clinic population described earlier, that is, white and mainly middle class and, therefore, not representative of the United Kingdom as a whole. However, this research was carried out in a University city, and the study cohort was not atypical for this individual clinic. Broadening settings to include views of different ethnic groups would be desirable as this issue is a weakness of much infertility research, with the exception of Culley's work with UK minority ethnic communities (Culley et al., 2007, 2013b). As the study was limited to a single UK site and only included treatment seekers, what is still unknown, therefore, is the experiences of couples who do not, for whatever reason, reach the clinic in the first place and this may be a consideration for future studies.

Our findings show that IVF is ubiquitous and that patients' introduction to it is often their first experience of privatised health care in the NHS. The well‐developed private provision and marketing of IVF (ANON, 2016) comes as a shock to many. While IVF is ‘sold’ as scientific, conception is far from guaranteed; buying an IVF cycle is not guaranteed to produce a pregnancy or live birth. There is still a great deal of uncertainty about the science of conception, as well as pregnancy loss (Freda et al., 2003).

In addition, buying an IVF cycle requires not only money but personal resources and investment and the process is stressful (Boivin et al., 1998), decision‐making is effortful at all stages, men can be marginalised in clinics, care is reduced to routinised and seemingly commercialised treatment plans, and there is little appetite for emotional care from providers. What is also clear is that experiences are prolonged and, despite great effort or the possibility of more treatment, this frequently leads nowhere, that is, there is little change to their status as involuntarily childless. This study indicates the ‘journey’ is not experienced as routine, even for patients who accept (or expect) the need for technology to assist them. Instead it is characterised by a recursive narrative of disequilibrium, rarely acknowledged in ongoing clinic–patient interactions.

This is the first longitudinal study to consider couples' experiences against a background of routine and normalised IVF. This study demonstrates that while current sociological research attends to new forms of family building, for example same sex couples using donor gametes, the majority of heterosexual couples using assisted reproduction have experiences which remain worthy of investigation. Currently, the normative status of IVF, at least in the context of health care, is at odds with the lived experiences of our participants.

## AUTHOR CONTRIBUTIONS

**Ginny Mounce:** Conceptualization (equal); investigation (lead); methodology (equal); project administration (lead); writing – original draft (lead). **Helen Allan:** Conceptualization (equal); investigation (supporting); methodology (supporting); supervision (lead); writing – review and editing (supporting). **Nicola Carey:** Conceptualization (supporting); formal analysis (supporting); methodology (supporting); project administration (equal); supervision (equal); visualization (supporting).

## Data Availability

The data that support the findings of this study are available from the corresponding author upon reasonable request.
